# Clinical value of 3'-deoxy-3'-[^18^F]fluorothymidine-positron emission tomography for diagnosis, staging and assessing therapy response in lung cancer

**DOI:** 10.1186/s13244-021-01026-1

**Published:** 2021-07-02

**Authors:** Bandar Alwadani, Sergio Dall’Angelo, Ian N. Fleming

**Affiliations:** 1 Diagnostic Radiology Department, College of Applied Medical Sciences, Jazan University, Al Maarefah Rd, POB 114, Jazan, 45142 Saudi Arabia; 2 Institute of Medical Sciences, University of Aberdeen, Aberdeen, AB25 2ZD UK

**Keywords:** Positron emission tomography, PET, Lung cancer, Fluorothymidine, [^18^F]FLT

## Abstract

Lung cancer has the highest mortality rate of any tumour type. The main driver of lung tumour growth and development is uncontrolled cellular proliferation. Poor patient outcomes are partly the result of the limited range of effective anti-cancer therapies available and partly due to the limited accuracy of biomarkers to report on cell proliferation rates in patients. Accordingly, accurate methods of diagnosing, staging and assessing response to therapy are crucial to improve patient outcomes. One effective way of assessing cell proliferation is to employ non-invasive evaluation using 3'-deoxy-3'-[^18^F]fluorothymidine ([^18^F]FLT) positron emission tomography [^18^F]FLT-PET. [^18^F]FLT, unlike the most commonly used PET tracer [^18^F]fluorodeoxyglucose ([^18^F]FDG), can specifically report on cell proliferation and does not accumulate in inflammatory cells. Therefore, this radiotracer could exhibit higher specificity in diagnosis and staging, along with more accurate monitoring of therapy response at early stages in the treatment cycle. This review summarises and evaluates published studies on the clinical use of [^18^F]FLT to diagnose, stage and assess response to therapy in lung cancer.

## Key points

- [^18^F]FLT is a useful surrogate biomarker of cell proliferation.
- [^18^F]FLT has higher specificity than [^18^F]FDG for diagnosing or staging lung cancer.
- [^18^F]FLT has lower sensitivity than [^18^F]FDG for diagnosing or staging lung cancer.
- [^18^F]FLT has good predictive values for assessing response to radiotherapy or chemoradiotherapy.
- [^18^F]FLT looks especially useful for assessing early response to targeted therapies.

## Background

Lung cancer (LC) remains the leading cause of cancer-related mortality worldwide, accounting for about 18.4 % of all cancer cases in 2018. It ranks first in mortality and incidence in men. In women it has the second highest mortality and the third highest incidence [1]. LC is subcategorised histologically into small cell lung cancer (SCLC) and non-small cell lung cancer (NSCLC), of which adenocarcinoma (ADC), squamous cell carcinoma (SqCC) and large cell lung carcinoma (LCC) represents almost 80 % of diagnosed cases [2]. These subcategories show different growth patterns and might be associated with different prognoses [3].

NSCLC can be stratified according to the tumour size, nodal involvement and metastases (TNM). SCLC is usually divided into limited or extensive disease, although the TNM system has been adopted by some clinical committees. These models, such as those provided by the Veterans Administration Lung Study Group and later modified by the International Association for the Study of Lung Cancer (IASLC), have proven useful to guide prognosis and patient stratification for therapy [4].

Uncontrolled cell proliferation is a fundamental hallmark of malignant tumour growth. The biomarker Ki-67 is considered the gold standard for assessment of cell proliferation due to the strong correlation between the cell proliferation rate and Ki67 expression in cells [5, 6]. However, it exhibits several potential drawbacks in the clinic, including the requirement for invasive collection of biopsies and possible sampling bias, due to poorly representative biopsies collected from heterogeneous tumours [7]. Over the past few decades, non-invasive positron emission tomography (PET) imaging has played an increasingly important role in LC management. [^18^F]Fluorodeoxyglucose ([^18^F]FDG) is the most widely used radiotracer in PET. It is a glucose analogue which monitors glucose metabolism, based on the concept that tumour cells take up significantly higher levels of glucose than normal tissues. It has been a beneficial adjunct in characterisation of intermediate solitary lung nodules and pre-treatment detection and staging of distant metastases [8]. However, [^18^F]FDG uptake is modulated by multiple signalling pathways, so is not selective enough to specifically assess changes in the cell proliferation rate. Moreover, a meta-analysis of [^18^F]FDG in LC showed its extreme heterogeneity in LC in pulmonary areas with inflammations [9].

**Fig. 1:**
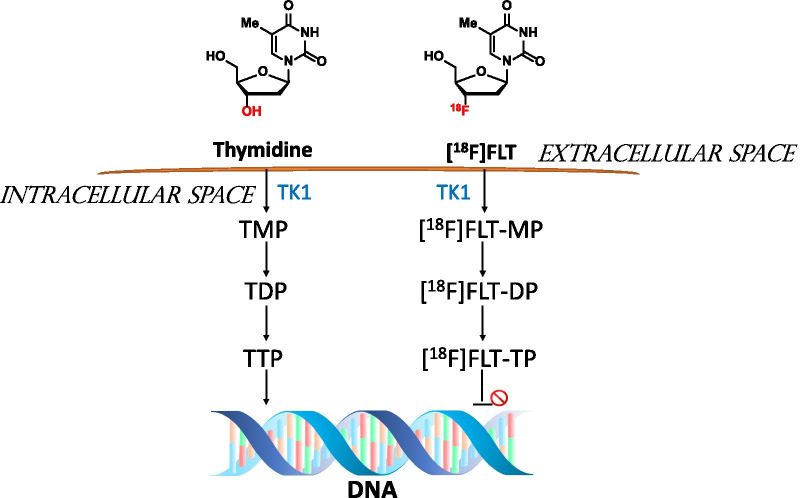
Summary of [^18^F]FLT uptake mechanism into cells. [^18^F]FLT sequestration in the cell after its phosphorylation by thymidine kinase 1 (TK1) into [^18^F]FLT monophosphate ([^18^F]FLT-MP), [^18^F]FLT diphosphate ([^18^F]FLT-DP) and [^18^F]FLT triphosphate ([^18^F]FLT-TP)

The first PET radiotracer introduced for in vivo proliferative imaging was ^11^C-labelled thymidine ([^11^C]thymidine); however, its rapid degradation with a half-life time of only 20 min is a major limitation [10]. 3'-deoxy-3'-**[**^18^F]fluorothymidine ([^18^F]FLT) was therefore subsequently employed, as this ^18^F-labelled thymidine analogue has a half-life of about 110 min. [^18^F]FLT is initially phosphorylated by cytosolic thymidine kinase 1 (TK1) into FLT-monophosphate and then subsequently further phosphorylated to make the diphosphate and triphosphate nucleotide (Fig. 1). TK1 is the principal enzyme that controls the rate of nucleotide recycling via the salvage pathway of DNA synthesis. After [^18^F]FLT is phosphorylated to the triphosphate analogue it cannot be further metabolised or incorporated into the DNA molecule. It is therefore trapped intracellularly as its high hydrophilicity means that it cannot readily cross the cell membrane. This cell sequestration of [^18^F]FLT is due to the substitution of the hydroxyl group at the 5’-end of thymidine, which is essential for ligation of DNA, with a fluorine-18 radionuclide. TK1 expression levels increase dramatically in proliferating cells, and there is close correlation between expression of the enzyme and the cell proliferation rate [11, 12]. Close correlation has also been observed between Ki-67 scoring and [^18^F]FLT uptake in many tumour types [5], including LC [6]. Therefore, [^18^F]FLT is a very plausible alternative to Ki-67 for assessing the proliferation rate in tumours. It also offers the advantages of avoiding the need to collect invasive biopsies and allows evaluation of proliferation heterogeneity across the entire tumour, minimising both patient discomfort and sampling errors. Moreover, [^18^F]FLT-PET is able to produce repeated 3D images for multiple cancer sites simultaneously, which is a distinct advantage for accurately assessing response to therapy. Alternative proliferation radiotracers have been also developed as direct biomarkers for DNA synthesis as they can be incorporated into DNA. This includes 2'-deoxy-2'-fluoro-5-methyl-1-β-d-arabinofuranosyluracil ([^18^F]FMAU) and 1-(2-deoxy-2-[^18^F]fluoro-β-d-arabinofuranosyl)-5-bromouracil ([^18^F]FBAU). However, these radiotracers are poor substrates for TK1 and show low uptake in highly proliferating tissue compared with [^18^F]FLT. This probably reflects their phosphorylation by mitochondrial thymidine kinase 2 (TK2) rather than TK1 [13], making [^18^F]FLT potentially superior to other proliferation radiotracers.

Relatively similar imaging protocols are followed in most [^18^F]FLT-PET scans. No specific preparations are required by patients although it is advantageous if they are instructed to drink up to 1 L water before imaging to stimulate tracer excretion from the renal calyces [14, 15]. The tracer dose administered ranges from 130 to 550 MBq. Most protocols employ static scans, although some more involved studies use dynamic scans to obtain more complex datasets. Subtly different scan timings and imaging parameters such as field of view and reconstruction techniques are also performed. For PET/CT, the patient is usually positioned supine, with arms raised to minimise beam hardening. Image evaluation is usually performed by two or more nuclear medicine physicians blinded to patient's data.

The aim of this literature review paper is to review the clinical value of [^18^F]FLT-PET proliferative imaging for diagnosis, staging and assessing response to therapy in LC. A secondary objective is to analyse the uptake values of [^18^F]FLT-PET in the various main LC sub-types in comparison with [^18^F]FDG and whether there is any difference between the two tracers in these sub-types. This overview will further enable clinicians to better understand the added value of [^18^F]FLT for clinical management of LC and its proliferative pattern in comparison with [^18^F]FDG in different LC sub-types.

## Materials and methods

### Search strategy

To identify all relevant publications, a systematic search of Scopus/PubMed databases was implemented from inception to 1st December 2020 using combinations of the following keywords: “positron emission tomography”, “PET”, “lung cancer”, “fluorothymidine” and “[^18^F]FLT”.

### Selection process

All potentially relevant publications were screened for eligibility. Initially, titles and abstracts were screened and if necessary, the full texts were scrutinised as well. Studies were included if they met the following criteria:

1. It was an original study that investigated the performance of [^18^F]FLT-PET for diagnosing, staging or assessing therapy response in LC patients;
2. It involved patients with suspected or confirmed malignant lesions;
3. The patients underwent chemotherapy, radiotherapy, chemoradiotherapy or targeted therapy; and
4. Clinical outcome was assessed.

Studies were excluded if they:

1. Only involved animal or in vitro studies;
2. Were not written in English or were not accessible in full text; and
3. Involved certain publication types: case reports, reviews, legal cases, editorials, letters, interviews, and comments.

### Statistical analysis of [^18^F]FLT uptake versus [^18^F]FDG uptake in lung cancer sub-types

Statistical analysis was performed using SPSS software. The specific research questions posed were as follows:

Is there any difference between the mean [^18^F]FDG and [^18^F]FLT uptake values in lung cancer patients? Null hypothesis was that there is no difference. Analysis was done using an unpaired students t-test.

Is there any different in mean radiotracer uptake values between the different lung cancer sub-types? This test was performed independently for [^18^F]FDG and [^18^F]FLT. Null hypothesis was that there is no difference. Statistical analysis was performed using a 1-way ANOVA with tukeys post hoc analysis and bootstrapping.

## Results

### Search results

**Fig. 2:**
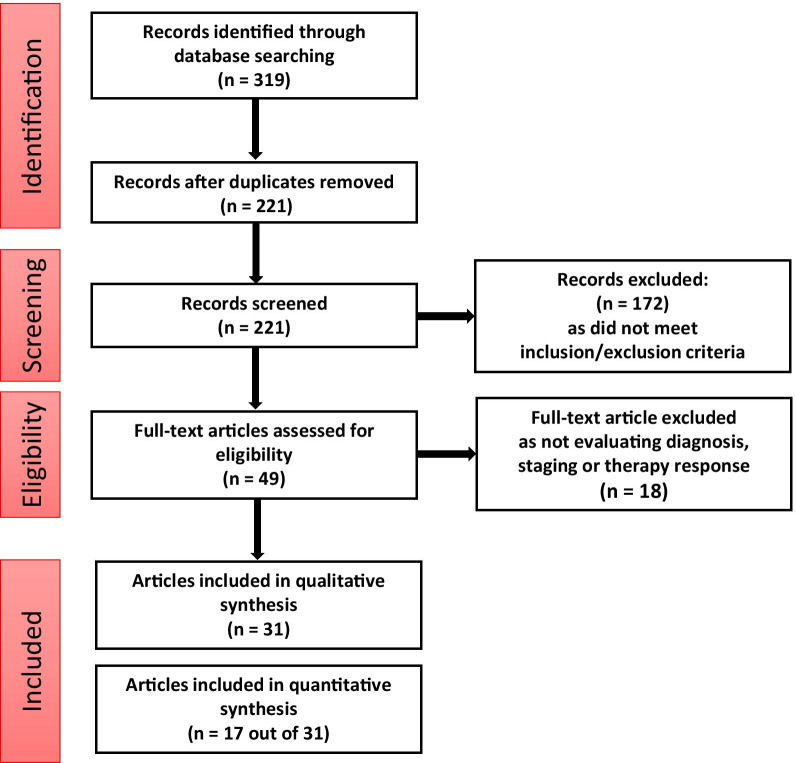
Flowchart of the search and selection process of studies

The literature search identified a total of 319 studies: 210 in Scopus and 109 in PubMed. After removing the studies that were found in both databases, 221 studies remained. Figure 2 presents the flow chart of the search and selection procedure. Out of 221, only 31 studies were included as eligible, and out of these 31 studies, 17 studies were included for quantitative synthesis. A summary of [^18^F]FLT-PET LC clinical studies and their findings is listed in Table 1 and 2.

**Table 1:** Summary of clinical studies evaluating diagnosis and staging and their findings in lung cancer based on [^18^F]FLT-PET proliferative imaging

Reference	n	[^18^F]FLT/[^18^F]FDG LC primary Tumour uptake (SUV)		[^18^F]FLT/[^18^F]FDG different LC histotypes uptake (SUVmax)	Purpose	TNM stage		Reference standard	Findings
Buck et al. 2002 [16]	30	Mean SUVmax = 5.2		SCLC (n = 1): 2.4	Diagnosis	TxN0M0 (n = 1); TxN0M1 (n = 4); T1N0M0 (n = 2); T1N1M0 (n = 2); T2N0M0 (n = 1); T2bN0M0 (n = 1); T2N1M0 (n = 4); T2N2Mx (n = 1); T2N2M1 (n = 1); T3N0M0 (n = 1); T3N3M0 (n = 1); T4N1M0 (n = 1); T4N2M0 (n = 2)		Histopathology Ki-67	[^18^F]FLT uptake was weak but easily detectable in Primary lesions. [^18^F]FLT is strongly correlated with Ki-67 in the malignant lesions
Vesselle et al. 2002 [27]	10	SUVmean: 3.84		LCC (n = 4): 5.29 SqCC (n = 3): 3.58 ADC (n = 2): 4.01	Diagnosis	NA		Histopathology Ki-67 S-phase fraction (SPF)	[^18^F]FLT SUVmax was correlated with Ki-67 and SPF (r = 0.78,* p* = 0.0043) and (r = 0.69,* p* = 0.03), respectively
Dittmann et al. 2003 [23]	16	[^18^F]FLT SUVmean: 4.0 [^18^F]FDG SUVmean: 6.9		NA	Diagnosis	NA		Histopathology Ki-67 [^18^F]FDG	[^18^F]FDG uptake was significantly higher than [^18^F]FLT uptake (*p* = 0.0006). [^18^F]FLT accurately detected thoracic lesions. Detection of lesions in the liver and the bone marrow was hampered with high physiological [^18^F]FLT uptake
Buck et al. 2003 [22]	26	[^18^F]FLT SUVmean: 1.8 [^18^F]FDG SUVmean: 4.1		[^18^F]FLT: SCLC (N = 1): 2.4 [^18^F]FDG: SCLC (N = 1): 12.7	Diagnosis	TxN0M0 (n = 1); TxN0M1 (n = 4); T1N0M0 (n = 1); T1N1M0 (n = 2); T2bN0M0 (n = 1); T2N1M0 (n = 4); T2N2Mx (n = 1); T3N0M0 (n = 1); T3N3M0 (n = 1); T4N2M0 (n = 2)		Histopathology Ki-67 [^18^F]FDG	[^18^F]FDG uptake was significantly higher than [^18^F]FLT uptake (*p* = 0.05)
Cobben et al. 2004 [15]	17	[^18^F]FLT Median SUVmax: 2.7 [^18^F]FDG Median SUVmax: 8		[^18^F]FLT: ADC (n = 4): 2.73 SqCC (n = 4): 2.55 [^18^F]FDG: ADC (n = 4): 7.38 SqCC (n = 4) = 9.83	Diagnosis Staging	TxN2/3M1 (n = 1); T1N0M1 (n = 1); T2N0M0 (n = 1); T2N2M0 (n = 1); T2N2M1 (n = 1); T2N2/3M1 (n = 1); T2N3M1 (n = 1); T4N0M1 (n = 2); T4N1M1 (n = 1); T4N2M0 (n = 3); T4N2M1 (n = 3)		Histopathology [^18^F]FDG	[^18^F]FDG uptake was significantly higher than [^18^F]FLT uptake (*p* = 0.012).. Staging by [^18^F]FLT-PET was correct for 8 of 17 patients
Buck et al. 2005 [17]	47	[^18^F]FLT SUVmean: 3.7 [^18^F]FLT SUVmax: 5.5 [^18^F]FDG SUVmean: 6 [^18^F]FDG SUVmax: 11		[^18^F]FLT: ADC (n = 8): 4.34 SqCC (n = 6): 6 LCC (n = 1): 5.7 SCLC (n = 1): 2.4 [^18^F]FDG: ADC (n = 8): 8.11 SqCC (n = 6): 6.5 LCC (n = 1): 12.5 SCLC (n = 1): 12.7	Diagnosis Staging	TcisN0M0 (n = 1); T1N0M0 (n = 2); T1N1M0 (n = 2); T1N3M0 (n = 1); T2N0M0 (n = 2); T2N1M0 (n = 5); T2N2M0 (n = 2); T3N0M0 (n = 1); T3N3M0 (n = 1); T4N1M0 (n = 1); T4N2M0 (n = 2); T4N3M0 (n = 1)		Histopathology [^18^F]FDG	[^18^F]FDG uptake was significantly higher than [^18^F]FLT uptake (*p* = 0.05). Clinical TNM stage was correctly identified in 67% of [^18^F]FLT scans, compared to 85% of [^18^F]FDG scans
Yap et al. 2006 [24]	22	[^18^F]FLT SUVmax: 1.6 [^18^F]FDG SUVmax: 3.1		[^18^F]FLT: ADC (n = 9): 1.11 SqCC (n = 3): 1.7 LCC (n = 1): 3.6 [^18^F]FDG: ADC (n = 9): 1.87 SqCC (n = 3): 3.53 LCC (n = 1) = 7	Diagnosis Staging	0 (n = 3); IA (n = 5); IB (n = 2); IIA (n = 1); IIIA (n = 2); IIIB (n = 2); IV (n = 7)		Histopathology Ki-67 [^18^F]FDG	[^18^F]FDG uptake was significantly higher than [^18^F]FLT uptake (*p* < 0.05) [^18^F]FLT overstaged (9%) of patients and understaged (36%) of patients whereas [^18^F]FDG overstaged (27%) of patients and understaged (14%) of patients
Yamamoto et al. 2007 [25]	18	[^18^F]FLT SUVmean: 3.6 [^18^F]FDG SUVmean: 8.5		[^18^F]FLT: ADC (n = 7): 3.43 SqCC (n = 5): 6.26 [^18^F]FDG: ADC (n = 7): 7.46 SqCC (n = 5) = 16.02	Diagnosis	T1N0M0 (n = 8); T1N2M0 (n = 1); T2N0M0 (n = 4); T2N1M0 (n = 2); T2N2M0 (n = 1); T3N2M0 (n = 1); T4N0M0 (n = 1)		Histopathology Ki-67 [^18^F]FDG	[^18^F]FDG uptake was significantly higher than [^18^F]FLT uptake (*p* < 0.0003)
Tian et al. 2008 [21]	55	[^18^F]FLT SUVmean: 3.54 [^18^F]FDG SUVmean: 8.13		[^18^F]FLT: ADC (n = 10): 3.72 SqCC (n = 2): 4.4 [^18^F]FDG: ADC (n = 10): 7.76 SqCC (n = 2): 9	Diagnosis with dual tracers [^18^F]FDG/[^18^F]FLT	NA		Histopathology [^18^F]FDG RECIST	The sensitivity and specificity of [^18^F]FDG and [^18^F]FLT were 87.5% and 58.97% and 68.75% and 76.92%, respectively. Improved sensitivity and specificity, 100% and 89.74%, respectively were shown with dual tracer [^18^F]FDG/[^18^F]FLT
Yamamoto et al. 2008 [19]	34	[^18^F]FLT SUVmean: 3.5 [^18^F]FDG SUVmean: 11.4		[^18^F]FLT: ADC (n = 16): 4.28 SqCC (n = 16): 5.57 SCLC (n = 2): 2.35 [^18^F]FDG: ADC (n = 16): 9.88 SqCC (n = 16): 12.72 SCLC (n = 2): 10.7	Diagnosis Staging	T1N1M0 (n = 1); T1N2M0 (n = 1); T2N0M0 (N = 10); T2N0M1 (N = 1); T2N1M1 (N = 2); T2N2M0 (N = 2); T3N0M0 (n = 1); T3N1M0 (n = 1); T3N2M0 (n = 2); T4N0M0 (n = 2)		Histopathology [^18^F]FDG	[^18^F]FDG uptake was significantly higher than [^18^F]FLT uptake (*P* < 0.0001). [^18^F]FLT showed same sensitivity and higher specificity and accuracy than [^18^F]FDG
Yang et al. 2010 [20]	31	[^18^F]FLT SUVmax: 4.2 [^18^F]FDG SUVmax: 7.7		[^18^F]FLT: ADC (n = 13): 3.8 SqCC (n = 11) = 5.7 [^18^F]FDG: ADC (n = 13): 8.4 SqCC (n = 11) = 8.1	Diagnosis Staging	T1N0 (n = 4); T1N1 (n = 5); T1N2 (n = 2); T2N0 (n = 6); T2N1 (n = 4); T2N2 (n = 2); T3N0 (n = 3); T3N1 (n = 3); T3N2 (n = 2)		Histopathology [^18^F]FDG	[^18^F]FLT showed significantly lower sensitivity (*p* = 0.031) for primary lesions. [^18^F]FLT showed better accuracy and specificity but lower sensitivity for nodal staging than [^18^F]FDG
Xu et al. 2016 [26]	14	[^18^F]FLT SUVmean: 4.96 [^18^F]FDG SUVmean: 8.12		[^18^F]FLT: ADC (n = 11): 5.14 SqCC (n = 3): 4.28 [^18^F]FDG: ADC (n = 11): 8.38 SqCC (n = 3): 7.13	Staging	T3N0M0 (n = 4); T3N1M0 (n = 3); T3N1M1 (n = 2); T4N0M0 (n = 1); T4N0M1 (n = 1); T4N1Mx (n = 1); T4N1M0 (n = 1); T4N1M1 (n = 1)		Histopathology [^18^F]FDG	[^18^F]FDG uptake was significantly higher than [^18^F]FLT uptake (*p* = 0.01). [^18^F]FLT showed better accuracy and specificity but lower sensitivity for nodal staging than [^18^F]FDG
Wang et al. 2016 [18]	55	[^18^F]FLT SUVmax: 2.9 [^18^F]FDG SUVmax: 6.8		NA	Diagnosis	NA		Histopathology Ki-67 [^18^F]FDG	[^18^F]FDG uptake was significantly higher than [^18^F]FLT uptake (*p* < 0.001*)* [^18^F]FLT showed lower sensitivity (68.75%) compared with 87.8% for [^18^F]FDG but higher specificity (77%) than [^18^F]FDG (59%) [^18^F]FLT can distinguish cancer from other solitary pulmonary nodules [^18^F]FDG showed an overlap in detecting cancer and tuberculosis

All of the reports in this table were single centre studies except Tian et al. 2008 [21]

n; Number of patients, NA; Not available, LC; Lung cancer, SCLC; Small cell lung cancer, NSCLC; non-small cell lung cancer, ADC; Adenocarcinoma, SqCC; Squamous cell carcinoma, LCC; Large cell lung carcinoma, TNM; Tumour, nodal involvement and metastases, PET; Positron emission tomography, [^18^F]FDG; [^18^F]fluorodeoxyglucose, [^18^F]FLT; 3'-deoxy-3'-[^18^F]fluorothymidine, SUV; Standardised uptake value, SUVmean; Mean standardised uptake value, SUVmax; Maximum standardised uptake value, SPF; S-phase fraction

**Table 2:** Summary of clinical studies evaluating therapy response and their findings in lung cancer based on [^18^F]FLT-PET proliferative imaging

Reference	n	[^18^F]FLT/[^18^F]FDG LC primary Tumour uptake (SUV)	[^18^F]FLT/[^18^F]FDG different LC histotypes uptake (SUVmax)	Purpose	TNM Stage		Reference standard	Findings
Frings et al. 2013 [28]	14	Variable uptake	NA	Study effect of pemetrexed-induced TS-inhibition on [^18^F]FLT uptake 4 h after pemetrexed administration	NA		Histopathology CT response	Changes in [^18^F]FLT uptake 4 h after pemetrexed administration were not predictive for tumour response, TTP or OS
Crandall et al. 2017 [29]	9	Mean SUVmax: Baseline: 5.6 ± 2.0 Post-cycle 1 (day 15–21): 4.8 ± 2.5 Post-cycle 2 (day 36–42): 4.4 ± 2.3	NA	Study early chemotherapeutic response in comparison with [^18^F]FDG	T1bN1M0 (n = 1); T1bN2M0 (n = 1); T2bN0M0 (n = 1); T2bN1M0 (n = 2); T3N0M0 (n = 2); T3N1M0 (n = 1); T3N2M0 (n = 1)		Histopathology CT response Ki-67 [^18^F]FDG	[^18^F]FLT did not show significant difference between anatomic responders and anatomic non-responders while [^18^F]FDG showed significant difference between these groups
McHugh et al. 2018 [30]	4	SUVmax from baseline in two patients:—64.7% and—54.3%	NA	Study the effect of dexamethasone on pemetrexed efficacy	NA		CT response	Decline of [^18^F]FLT uptake from baseline, with a variable response between individual tumour lesions
Vera et al. 2011 [31]	5	Mean SUVmax: Baseline: 4.7 During therapy: 2.2	[^18^F]FLT: Baseline: ADC (n = 2): 4.64 SqCC (n = 2): 3.86 During therapy: ADC (n = 2): 2.24 SqCC (n = 2): 2.13 [^18^F]FDG: Baseline: ADC (n = 2): 7.76 SqCC (n = 2): 6.36 During therapy: ADC (n = 2): 3.24 SqCC (n = 2): 5.38	Study radiotherapy responses in comparison with [^18^F]FDG and F-miso	TXN3 (n = 1);T2N2 (n = 1);T3N2 (n = 1);T4N2 (n = 1); T4N3 (n = 1)		Histopathology	A significant decrease in SUVmax during radiotherapy was observed for [^18^F]FLT and [^18^F]FDG but not for F-miso
Saga et al. 2011 [32]	20	Mean SUVmax: baseline: 1.54 3 months posttherapy: 0.35	NA	Longitudinal study of the responses to carbon-ion radiotherapy	T1N0M0 (n = 9); T2N0M0 (n = 11)		Histopathology CT response	[^18^F]FLT uptake significantly decreased after treatment Basline [^18^F]FLT uptake of patients who developed recurrence and who died of LC were significantly higher than that of patients who did not (*p* = 0.008 and 0.007*)*
Trigonis et al. 2014 [33]	16	SUVmean: baseline: 2.2 ± 0.7 Response value: 1.6 ± 0.4 SUVmax: baseline: 5.3 ± 2.0 Response value: 4.1 ± 1.4	NA	Study early radiotherapy response and test–retest variability	NA		Histopathology CT response	SUVmean decreased by 25% in the absence of volumetric change (*p* = 0.0001) after 5–11 radiotherapy fractions. Larger decrease of 40% was shown in metastatic nodes with 31% decrease in volume (*p* < 0.0001). Similar findings for SUVmax were found. SUVmean reproducibility (standard deviation [SD]: 8.9%) in primary tumours was better than SUVmax reproducibility (SD: 12.6%)
Everitt et al. 2009 [34]	5	SUVmax: mean reduction of 0.58 × baseline	[^18^F]FLT: Baseline: ADC (n = 1): 5.3 SqCC (n = 2): 8.25 LCC (n = 2): 5.15 [^18^F]FDG: Baseline: ADC (n = 1): 15 SqCC (n = 2): 17.15 LCC (n = 2): 9.4	Establish [^18^F]FLT assessment of cell proliferation during chemoradiotherapy	T2N0M0 (n = 1); T2N2M0 (N = 1); T2N3M0 (n = 1); T3N2M0 (n = 2)		Histopathology	Decline of [^18^F]FLT uptake after chemotherapy in epithelial cancers and bone marrow (radiosensitive tissue)
Everitt et al. 2014 [35]	20	[^18^F]FLT Median SUVmax: baseline: 6; wk2: 3; wk4: 2 [^18^F]FDG Median SUVmax: baseline: 14; wk2: 10; wk4:10	NA	Study early chemo-radiotherapeutic response in comparison with [^18^F]FDG	NA		Histopathology CT response	[^18^F]FLT is a more sensitive tracer for early treatment response than [^18^F]FDG
Everitt et al. 2017 [36]	60	NA	NA	Study relationship between chemo-radiotherapeutic responses and clinical outcomes in comparison with [^18^F]FDG	NA		[^18^F]FDG	Stable uptake of [^18^F]FLT at 2 wk after therapy was associated with longer OS and PFS compared with patients whose tumours demonstrated reduced or absent [^18^F]FLT uptake Paradoxical association between changes in [^18^F]FLT uptake and clinical outcomes. This could be due to weakening tumourocidal effect of radiotherapy with inhibitory effect of possibly chemotherapy (carboplatin and paclitaxel)
Yang et al. 2012 [37]	68	Mean SUVmax: 4.1	Baseline: ADC (n = 30): 3.8 SqCC (n = 28): 5	Study antiangiogenic therapy responses in comparison with MVD	NA		Histopathology Ki-67 MVD	[^18^F]FLT uptake was significantly correlated with MVD as reflected by CD105-MVD (r = 0.633,* p* = 0.000). Patients with lower [^18^F]FLT uptake and CD105-MVD values had significantly higher median survival times than patients with higher [^18^F]FLT uptake and CD105-MVD values (*p* = 0.046)
Scarpelli et al. 2018 [38]	14	Change in SUVmax: during angiogenic therapy: −11% After combination chemotherapy: −44%	NA	Study the sequential responses of angiogenic therapy followed by chemotherapy	NA		Histopathology	[^18^F]FLT uptake significantly decreased during therapy (*p* = 0.04) and after combination chemotherapy (*p* = 0.03)
Scarpelli et al. 2018 [39]	33	NA	NA	Study the sequential responses of angiogenic therapy followed by chemotherapy	NA		Histopathology	Tumour cell proliferation and vasculature were decreased 2 weeks after therapy and increased one week after therapy break
Sohn et al. 2008 [40]	31	Mean SUVmax decline, 7 days posttherapy:—36.0% for responders versus -10.1% for nonresponder (-10.9% was ued as cutoff)	NA	Study early EGFR-TKI (gefitinib) therapy responses to predict clinical outcome	IV (n = 28)		CT response	[^18^F]FLT uptake significantly decreased 7 days after gefitinib therapy in responders. Responders had significantly longer TTP (*p* = 0.03) than non-responders
Mileshkin et al. 2011 [41]	51	[^18^F]FLT SUVmax: baseline: 2.5 [^18^F]FDG SUVmax: 5.63	NA	Study early EGFR-TKI (erlotinib) therapy responses to predict clinical outcome in comparison with [^18^F]FDG	NA		CT response	PFS was predicted by both [^18^F]FLT and [^18^F]FDG 2wks and 8wks after therapy. OS was predicted by both tracers 8wks after therapy but by [^18^F]FDG only 2wk after therapy
Zander et al. 2011 [42]	34	[^18^F]FLT Mean SUVpeak: baseline: 3.47 week2: 3.12 [^18^F]FDG Mean SUVpeak: baseline: 6.94 week2: 6.03	NA	Study early EGFR-TKI (erlotinib) therapy responses to predict clinical outcome in comparison with [^18^F]FDG	NA		CT response	[^18^F]FLT predicted PFS but not OS 6 wks after therapy, while [^18^F]FDG predicted PFS, OS 6 wks after therapy
Kahraman et al. 2011 [43]	30	NA	NA	Study early EGFR-TKI (erlotinib) therapy responses to predict clinical outcome in comparison with [^18^F]FDG	NA		CT response	Both [^18^F]FLT and FDG predicted PFS 1wk and 6wk after therapy
Scheffler et al. 2013 [44]	40	Mean SUVmax,pretherapy: 3.0	[^18^F]FLT: Baseline: ADC (n = 29): 3.18 SqCC (n = 6): 3.53 LCC (n = 1): 1.9 [^18^F]FDG: Baseline: ADC (n = 29): 7.09 SqCC (n = 6) = 6.38 LCC (n = 1): 6.6	Study the prognostic value of baseline uptake in patients treated with EGFR-TKI (erlotinib) in comparison with [^18^F]FDG	NA		Histopathology Ki-67	[^18^F]FLT to prognostically stratify NSCLC patients treated with erlotinib as patients with low uptake had significantly longer survival times (*p* = 0.027) than patients with high uptake
Bhoil et al. 2014 [45]	15	NA	NA	Study TKI (gefitinib or erlotinib) therapy responses to predict clinical outcome in comparison with [^18^F]FDG	NA		CT response	Neither OS nor PFS were correlated with [^18^F]FLT but both correlated with [^18^F]FDG

All of the reports in this table involved single centre studies except Crandall et al. 2017 [29] and Mileshkin et al. [41]

n; Number of patients, NA; Not available, LC; Lung cancer, SCLC; Small cell lung cancer, NSCLC; non-small cell lung cancer, ADC; Adenocarcinoma, SqCC; Squamous cell carcinoma, LCC; Large cell lung carcinoma, PET; Positron emission tomography, [^18^F]FDG; [^18^F]fluorodeoxyglucose, [^18^F]FLT; 3'-deoxy-3'-[^18^F]fluorothymidine, SUV; Standardised uptake value, SUVmean; Mean standardised uptake value, SUVmax; Maximum standardised uptake value, SUVpeak; Standardised uptake value peak, PFS; Progression free survival, TTP; Time to progression, OS; Overall survival, MVD; Microvessel density, EGFR-TKI; Epidermal growth factor receptor- tyrosine kinase inhibitor

### Diagnosis studies

The past two decades have witnessed a growing attention to [^18^F]FLT as a potential diagnostic to aid LC patient management. Imaging with [^18^F]FLT has shown added benefit to diagnostic accuracy. Buck et al. (n = 30) and Vessel et al. (n = 10) introduced the clinical potential of [^18^F]FLT in LC by demonstrating that the tracer accumulates primarily in malignant lesions, with minimal uptake in benign lesions. The latter study also showed [^18^F]FLT maximum standardised uptake value (SUVmax) strongly correlates with both Ki-67 index and S-phase fraction (SPF), measured using flow cytometry [14, 16]. Further, Buck et al. observed mean SUVmax of 5.2 in malignant tumours compared with no apparent uptake in benign tumours [16]. This observation was supported by another study which showed primary tumours exhibiting almost identical [^18^F]FLT uptake values [17]. Furthermore, Wang et al. (n = 55) observed significantly higher [^18^F]FLT uptake (*p* < 0.05) in LC lesions compared with all other solitary pulmonary nodules, including tuberculosis, inflammatory and benign lesions, whereas tuberculosis showed similar [^18^F]FDG-SUVmax (6.9) values to LC (6.8) [18]. In multiple comparative studies with [^18^F]FDG [18–21], higher specificity (ability to exclude non-malignant lesions) was presented using [^18^F]FLT. The imaging specificity of [^18^F]FLT ranges from about 77% to 99% versus about 50% to 84% for imaging with [^18^F]FDG in a total number of 195 patients of all study cohorts combined.

On the other hand, the relatively low uptake of [^18^F]FLT adversely affects its detection ability in comparison with [^18^F]FDG. For example, Buck et al. exhibited the significantly lower uptake of [^18^F]FLT compared with [^18^F]FDG (*p* < 0.05) [17]. Similar significant differences between [^18^F]FLT and [^18^F]FDG uptakes were also reported in a number of other studies [18–20, 22–26]. Also, lower sensitivity was shown with [^18^F]FLT-PET compared with [^18^F]FDG-PET. The sensitivity of [^18^F]FLT-PET range was only 65%–83% versus 85%–97% for [^18^F]FDG-PET in 195 patients combined from the mentioned studies. Given the higher specificity of [^18^F]FLT-PET but lower sensitivity in comparison with [^18^F]FDG-PET, it was proposed that a combination of both [^18^F]FLT and [^18^F]FDG-PET could potentially provide better diagnostic performance than the individual tracers. Indeed, this strategy was conducted by a multicentre study consisted 55 patients with suspected LC [21], resulting in sensitivity improving from 87% (with [^18^F]FDG alone) to 100% and specificity increasing from 77% (with [^18^F]FLT alone) to approximately about 90%.

### Proliferative imaging of lung cancer sub-types with [^18^F]FLT versus [^18^F]FDG

**Fig. 3:**
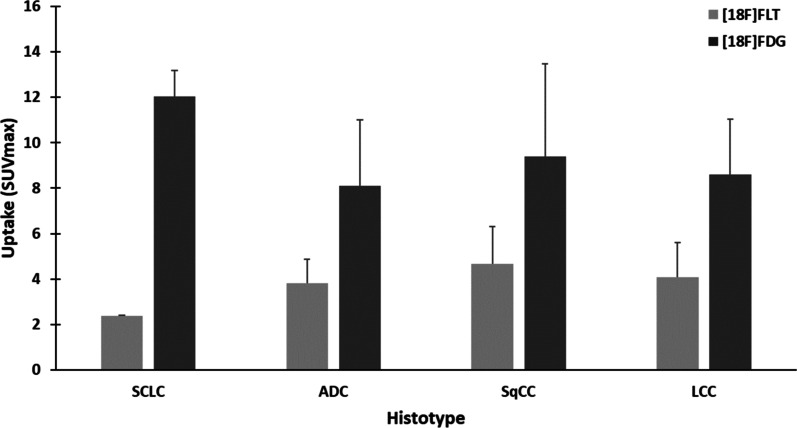
Histological association of [^18^F]FLT SUVmax and [^18^F]FDG SUVmax in different histotypes of LC

Histological uptake in this review showed that [^18^F]FLT-SUVmax is significantly lower than [^18^F]FDG-SUVmax across all LC histotypes (*p* < 0.0001), with different uptake values in the various histotypes. [^18^F]FDG-SUVmax, as illustrated in Fig. 3, demonstrated the greatest mean SUVmax in SCLC (12.03) followed by SqCC (9.38), LCC (8.60), and ADC (8.10). A comparison of [^18^F]FDG-SUVmax detected no significant difference in the 4 sub-types of LC (*p* > 0.05), although the statistical power of this analysis is limited by the number of SCLC studies performed to date.

The highest [^18^F]FLT-mean SUVmax was in SqCC (4.65) followed by LCC (4.07), ADC (3.82), and SCLC (2.39). A comparison of [^18^F]FLT-SUVmax between the different histotypes observed that [^18^F]FLT-SUVmax was significantly lower in SCLC than in SqCC (*p* < 0.05). Uptake patterns with both tracers in NSCLC sub-types are consistent with the proliferative patterns demonstrated by Ki-67 in a previous study in the different sub-types of NSCLC histology [26].

### Staging studies

Staging of LC is another clinical application that has been evaluated in several studies. In general, [^18^F]FLT-PET has failed to demonstrate better TNM staging than [^18^F]FDG-PET. Initially, a preliminary study in 2004 showed that, in comparison with clinical TNM staging, [^18^F]FLT may be limited; [^18^F]FLT staging identified 9 out of 17 patients incorrectly [15]. Buck et al. compared [^18^F]FLT-PET with [^18^F]FDG-PET for staging 47 patients with suspected malignant nodules. The clinical TNM stage according to histopathology was correctly identified in 67% of [^18^F]FLT scans compared with 85% of [^18^F]FDG scans [17]. Furthermore, Yap et al. (n = 22) also evaluated [^18^F]FLT staging in comparison with [^18^F]FDG, taking histopathology data as a reference standard. According to histopathology, 3 patients were disease free, 10 patients were at early resectable stages and 7 were at late inoperable stages. [^18^F]FLT overstaged two patients (9%) and understaged eight patients (36%) whereas [^18^F]FDG overstaged six patients (27%) and understaged three patients (14%) [46]. Likewise, Yang et al. (n = 31) observed that more patients are understaged with [^18^F]FLT and more patients are overstaged with [^18^F]FDG. In this study, understaged patients represent 16% with [^18^F]FLT and 6% with [^18^F]FDG and overstaged patients represent 6% with [^18^F]FLT and 16% with [^18^F]FDG [20].

In addition, sensitivity, specificity and accuracy of [^18^F]FLT for nodal involvement were evaluated in 5 studies in comparison with [^18^F]FDG. The data showed clear differences between the two tracers. Buck et al. (n = 47) compared [^18^F]FLT-PET staging with [^18^F]FDG-PET staging in 47 patients with suspected malignant nodules and found that the clinical TNM stage was correctly identified in 67% of [^18^F]FLT scans compared with 85% of [^18^F]FDG scans [17]. Although both [^18^F]FLT and [^18^F]FDG showed 100% specificity for staging of lymph nodes in this study, the sensitivity and accuracy of [^18^F]FDG was higher (77% and 83%) than those of [^18^F]FLT (53% and 67%). Another study of 31 NSCLC patients [20] demonstrated even greater specificity and accuracy for [^18^F]FLT in staging lymph nodes than [^18^F]FDG but lower sensitivity than that of [^18^F]FDG. The sensitivity, specificity and accuracy for [^18^F]FLT were 65%, 85% and 93%, respectively, whereas the equivalent [^18^F]FDG values were 98%, 84% and 84%, respectively [20]. In contrast, Yamamoto et al. (n = 34) showed the same sensitivity (57%) for both tracers but higher specificity and accuracy (93% and 85%) for [^18^F]FLT versus 78% and 74% for [^18^F]FDG [19]. Moreover, Xu et al. (n = 14) reported better sensitivity, specificity and accuracy for [^18^F]FLT (85%, 93% and 85%, respectively) than for [^18^F]FDG (93%, 78% and 84%) [26]. However, taken together, the weighted mean for sensitivity, specificity and accuracy in all of these studies for [^18^F]FLT are 61%, 94%, and 80%, respectively, and for [^18^F]FDG are 79%, 88% and 80%, respectively.

### Studies assessing therapy response

#### Chemotherapy

Three studies have investigated the potential of [^18^F]FLT for assessing chemotherapy and the findings were not promising. Frings et al. [28] showed that change in [^18^F]FLT uptake 4 hours after treatment with pemetrexed, an inhibitor of thymidylate synthase, was neither significantly correlated with time to progression (TTP) nor with overall survival (OS) in 14 patients (*p* = 0.96 and 0.43, respectively). Likewise, McHugh et al. [30] investigated the effect of dexamethasone, a drug used to mitigate side effects of chemotherapy, on pemetrexed efficacy. They demonstrated that [^18^F]FLT could critically detect heterogeneity in dexamethasone sensitivity between tumours within individual patients. A comparison with [^18^F]FDG-PET was performed in 9 patients treated with neoadjuvant chemotherapy in a former study and categorised according to tumour size assessed by computed tomography [29]. Using [^18^F]FDG, anatomic responders showed significantly lower uptake than anatomic non-responders 2-3 weeks after treatment, while no significant difference was found between these subgroups using [^18^F]FLT.

#### Radiotherapy

Preliminary data of studies that evaluated response to radiotherapy is more promising. Vera et al. [31] demonstrated significant changes in SUVmax in 5 patients treated with 46 Gy radiotherapy. This decline of [^18^F]FLT uptake was supported by another study (n = 20) which tested correlation with clinical results after treatment with carbon ion radiotherapy [32]. Baseline SUVmax was predictive of response as patients who died or developed recurrence had significantly more [^18^F]FLT uptake than those did not (*p* = 0.007 and* p* = 0.008, respectively). Furthermore, primary tumours showed decreased SUVmean and SUVmax despite the absence of morphological change [33].

#### Chemoradiotherapy

Three studies have investigated the potential of [^18^F]FLT for assessing response to chemoradiotherapy. Response to chemoradiotherapy was firstly assessed in a small study (n = 5), which observed decreased [^18^F]FLT uptake in primary tumours after therapy [34]. A subsequent study (n = 20) found that [^18^F]FLT was more sensitive than [^18^F]FDG in assessing response to radical chemo-radiation [35]. Median SUVmax were 14 and 6 at baseline and 10 and 3 two weeks post-therapy for [^18^F]FDG and [^18^F]FLT, respectively. The same group also studied correlation with clinical outcome in a larger cohort (n = 60) treated with 60 Gy radiotherapy combined with carboplatin and paclitaxel or cisplatin (32). Stable disease, as assessed using RECIST criteria, was associated with longer OS and progression-free survival (PFS) than patients with complete or partial response. This paradox could be ascribed to the weakening of tumouricidal effect of chemotherapy by suppressed proliferation [36].

#### Targeted therapy

Multiple studies have found high potential of [^18^F]FLT to predict LC response to targeted therapy. Yang et al. analysed the prediction of response to anti-angiogenic agents in correlation with microvessel density (MVD) [37]. [^18^F]FLT was correlated significantly with MVD as reflected by CD105-MVD as well as clinical outcomes. Longer median survival times were observed in patients who had [18F]FLT false negative results (*p* = 0.012) and in patients with lower CD105-MVD (*p* = 0.046). Moreover, the ability of [^18^F]FLT to monitor pharmacodynamic effect therapy was examined by Scarpelli et al. with static (n = 14) and dynamic (n = 33) PET [38, 39]. In the static PET study, SUVmax was decreased −11% in cycle 1 treatment with the anti-angiogenic x-82. After administration of cycle 2 x-82 combined with the chemotherapy docetaxel on day 21, SUVmax was greatly decreased to −44% (34). Decline of cell tumour proliferation and vasculature were also exhibited 2 weeks after treatment with axitinib followed by an increase in washout period during the week of treatment break [39].

**Fig. 4:**
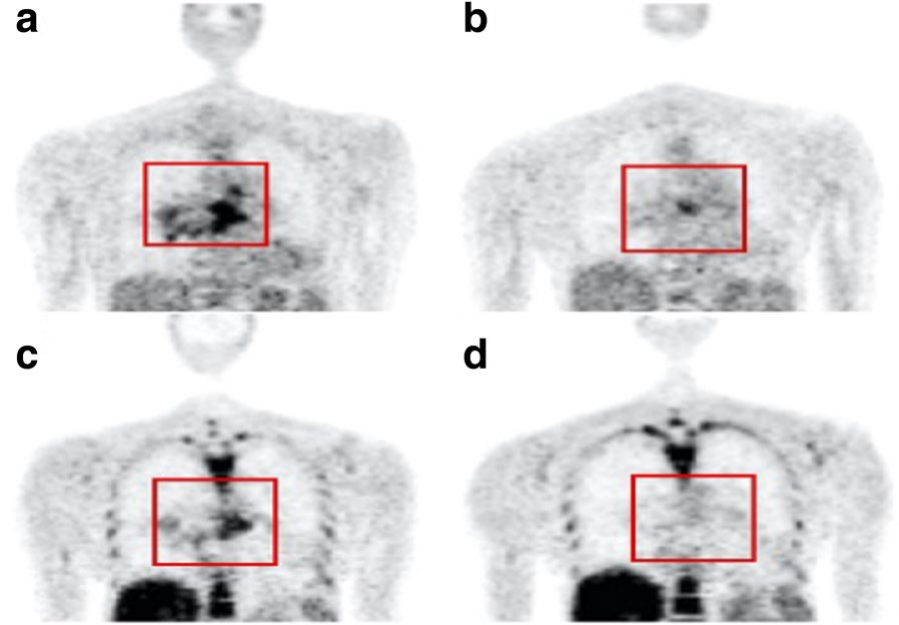
Evaluating response to erlotinib therapy with [^18^F]FDG-PET and [^18^F]FLT-PET. [^18^F]FDG-PET (**a**) and [^18^F]FLT-PET (**c**) before start of treatment, and [^18^F]FDG-PET (**b**) and [^18^F]FLT-PET (**d**) after 1 week of treatment with erlotinib. [^18^F]FDG uptake is higher than [^18^F]FLT in the baseline scans. Erlotinib treatment decreases uptake of both radiotracers. The [^18^F]FLT scan after therapy shows minimal uptake indicating that erlotinib is effectively inhibiting proliferation. Reprinted with permission from JNM. This research was originally published in JNM. Kahraman et al. [43]

Assessment of epidermal growth factor receptor-tyrosine kinase inhibitors (EGFR-TKI) response with [^18^F]FLT is also an active area of research. Typical scans of [^18^F]FLT uptake before and after EGFR-TKI therapy in a LC patient responding to therapy are shown in Fig. 4. The value of [^18^F]FLT in this role is due to well-established clinical benefits of EGFR-TKI, as they are being used as first-line therapy in select NSCLC patients [47]. Three studies investigated the performance of [^18^F]FLT in assessing tumour progression in correlation with tumour size measured by computed tomography. Sohn et al. utilised [^18^F]FLT to predict response to gefitinib in 31 adenocarcinoma patients [40]. A significant difference (*p* < 0.001) was shown between responders and non-responders. Mileshkin et al. also evaluated [^18^F]FLT and [^18^F]FDG to monitor response in 51 NSCLC patients 2 and 8 weeks after treatment with erlotinib (first generation EGFR-TKI drug). 4 responders were assigned by computed tomography; all of them were classified responders by [^18^F]FDG and 3 of them by [^18^F]FLT [41]. In contrast, Zander et al. found that tumour size measurements correlated with changes in [^18^F]FDG uptake (*p* < 0.05) but not with [^18^F]FLT (*p* > 0.05) 6 weeks after erlotinib therapy [42]. Moreover, further clinical assessment of [^18^F]FLT role for early monitoring of response to newer generations of EGFR-TKI agents is still required in order to better understand their associated mechanisms of responsiveness and resistance [48].

The predictive role of clinical outcome using [^18^F]FLT uptake was also studied in EGFR-TKI studies and showed positive results. Sohn et al. demonstrated that [^18^F]FLT responders showed significantly longer TTP (*p* = 0.0041) than non-responders. Further, Scheffler et al. (n = 40) observed that [^18^F]FLT could prognostically stratify NSCLC patients treated with erlotinib, as patients with low uptake had significantly longer survival times (*p* = 0.027) than patients with high uptake [44]. Moreover, apart from one study with a small patient cohort (n = 15) [45], [^18^F]FLT uptake was correlated with PFS in multiple independent studies with larger cohorts [41–43]. However, in most studies OS did not correlate with [^18^F]FLT uptake [42, 45].

## Discussion

This paper has reviewed the literature of clinical [^18^F]FLT applications in LC, and compared the uptake values of [^18^F]FLT and [^18^F]FDG in the various main LC sub-types. The review clearly shows that [^18^F]FLT is able to provide useful data to help diagnose, stage and monitor therapy response in LC. However, the value of using [^18^F]FLT-PET in some of these roles is stronger than in others. The paper also outlines some novel observations about the studies performed to date and provides some recommendations that could help to guide the design of prospective clinical studies with [^18^F]FLT in LC.

For diagnosis and staging, [^18^F]FLT showed better specificity and discriminative value between inflammatory and malignant LC than [^18^F]FDG. The superior specificity of [^18^F]FLT can be attributed to its highly selective uptake in highly proliferating (i.e. malignant) cells, providing better differentiation of malignant from benign tumours. [^18^F]FDG exhibits a slightly lower specificity than [^18^F]FLT due to the high metabolic activity of some benign lesions and inflammatory cells which use glucose as the main substrate for energy production [19]. On the other hand, [^18^F]FLT shows significantly lower uptake and, thus, sensitivity than [^18^F]FDG in LC. The lower sensitivity of [^18^F]FLT is in concordance with a previous systematic review and meta-analysis study which also deduced that [^18^F]FLT is a more specific but less sensitive radiotracer than [^18^F]FDG [49]. The low sensitivity of [^18^F]FLT appears to be the reason behind its understaging of tumours compared to [^18^F]FDG. This limitation makes it unable to replace [^18^F]FDG in these roles, although it may be able to complement [^18^F]FDG in some situations. The relatively low uptake of [^18^F]FLT in cancer cells is partly due to the fact that cells do not express a specialised transporter to facilitate its transport across the cell membrane; [^18^F]FLT either enters cells via the general nucleoside transporter hENT1 or via passive diffusion [50]. Furthermore, highly proliferating cells such as bone marrow act as a sink, reducing the amount of [^18^F]FLT available in the body to be taken up by tumours [51]. In contrast, cancer cells tend to take up relatively high levels of [^18^F]FDG. This is due to multiple factors. Firstly, they express specialised receptors such as GLUT1, which facilitate the active transport of glucose or [^18^F]FDG across the cell membrane, and partly due to the Warburg effect, which results in relatively high glucose metabolism in tumour cells. Secondly, whilst most cancer cells are highly metabolically active only a proportion of them are proliferating at any time. Finally, inflammatory cells are usually present in malignant tumours, which will further increase [^18^F]FDG uptake in regions of interest.

One interesting suggestion is that using [^18^F]FLT and [^18^F]FDG-PET in combination could potentially provide better diagnostic performance than the individual tracers. Indeed, this strategy showed higher sensitivity and specificity in diagnosing pulmonary lesions [21]. However, this benefit is offset by the higher costs associated with performing two PET scans and the increased radiation burden imposed by this dual tracer approach. There is an effective dose equivalent of 0.031 mSv/MBq and 0.029mSv/MBq for [^18^F]FLT and [^18^F]FDG, respectively [52]. When 2 doses of 400 MBq were administered by Tian et al. [21] this resulted in a total radiation dose per patient of approximately 24 mSv (12.4 from [^18^F]FLT + 11.6 from [^18^F]FDG) within a week. Owing to the increased radiation dose, the dual tracer strategy should be valued favourably only for patients with an equivocal diagnosis where the benefit of more confident diagnosis could brought to the patient, taking into account the patent's clinical characteristics.

An analysis of LC studies to date confirmed that there is significantly lower [^18^F]FLT uptake than [^18^F]FDG uptake in all histotypes tested (Fig. 3). This result was expected given the defined uptake mechanism for [^18^F]FDG in tumour cells combined with the Warburg effect, which results in tumour cells utilising high amounts of glucose. A comparison of [^18^F]FDG-SUVmax detected no significant difference between the 4 sub-types of LC (*p* > 0.05). However, the statistical power of this analysis is limited by the number of SCLC studies performed to date and the relative high variation in uptake values between studies in the other tumour types. Publication of additional LC datasets is required to confirm if there is any clear differences in [^18^F]FDG uptake between LC sub-types. In contrast, a comparison of [^18^F]FLT-SUVmax between the different histotypes observed that [^18^F]FLT-SUVmax was significantly lower in SCLC than in SqCC (*p* < 0.05). This is a new observation which suggests that there may be something subtly different between these LC sub-types. There are 3 potential straightforward explanations for this observation. The first possibility is that SCLC proliferates at a slower rate. This seems unlikely given that SCLC is known to be an aggressive sub-type that grows quickly. The second possibility is that there is less cellular [^18^F]FLT uptake into SCLC, either because these tumours have limited access to [^18^F]FLT in the blood or because less radiotracer can cross the plasma-membrane, perhaps suggesting that the cells may express fewer hENT transporters. The third possibility is that SCLC could rely more than SqCC on the *de novo* pathway to synthesise nucleotides rather than using the salvage pathway [53], resulting in less [^18^F]FLT uptake into cells via hENT transporters. To our knowledge neither of the latter possibilities have been investigated to date.

Evaluating the potential of [^18^F]FLT-PET for assessing early response to therapy is a logical step, given the clear utility of [^18^F]FDG-PET in this role in LC. The rationale for using [^18^F]FLT-PET to assess therapies that selectively affect proliferation is especially compelling, as these treatments may inhibit proliferation without affecting either metabolic rate or causing tumour shrinkage [54]. Only 4 out of the 18 LC response assessment studies identified in this review did not observe some utility for [^18^F]FLT in this role [28, 29, 36, 42]. One of these studies [28] assessed a chemotherapeutic agent that is antagonist to thymidylate synthase. This may impact TK1, resulting in poor correlation between [^18^F]FLT uptake and clinical outcomes [53]. Whilst another study [29], which studied platinum-based chemotherapy, showed no correlation with CT response. The reason for this discrepancy is unknown. These findings suggest that the complex effect of cytotoxic therapeutics on cell signalling pathways needs to be fully considered to understand how each agent may affect [^18^F]FLT uptake into cells. Indeed, a previous systematic review that evaluated the role of [^18^F]FLT as a measure of therapy response in different tumours also concluded that [^18^F]FLT-PET is not as useful as [^18^F]FDG-PET for assessing chemotherapeutic response in NSCLC [55]. However, studies evaluating these types of therapies are still scarce and findings from more studies with larger patient cohorts might be more conclusive. A chemoradiotherapy study which employed [^18^F]FLT-PET to evaluate therapy response observed that stable disease measurements compared with complete or partial response based on CT was associated with longer OS and progression-free survival (PFS) [36]. This paradox could be ascribed to the weakening of tumouricidal effect of chemotherapy by suppressed proliferation [36]. Finally, a study evaluating [^18^F]FLT-PET to assess EGFR-TKI response found no correlation with CT measurements [42]. However, this observation may be due to utilisation of size measurements to assess EGFR-TKI; change in tumour size may be delayed or may not occur at all with this type of therapy, creating a discrepancy between these two different metrics [54].

The most promising application for [^18^F]FLT is assessing response to targeted agents that selectively inhibit cell proliferation. In this role [^18^F]FLT is utilised as a precise tool for detecting the effects of antiproliferative therapies. Apart from one study with a small patient cohort (n = 15) [45], [^18^F]FLT uptake correlated with PFS in multiple independent studies with larger cohorts [41–43]. Interestingly, in most studies OS did not correlate with [^18^F]FLT uptake [42, 45]. This may be due to the subtly different metrics captured by PFS and OS. The OS statistics may be somewhat compromised by the limited number of patients recruited in most of these studies and/or the limited follow up time. Indeed, the study with the largest patient group (n = 51) and follow up time observed correlation between [^18^F]FLT uptake and OS 8 weeks after treatment; the longest follow up time in other studies was only 6 weeks [41, 42]. Although OS is a gold standard measure to demonstrate clinical efficacy, larger patient numbers and longer follow up are required to establish [^18^F]FLT as a reliable measure of this endpoint [56]. PFS is more advantageous than OS in that it assesses both stable disease and responsive disease. This renders it a more reasonable clinical measure for targeted therapies, which often benefit patients mainly through prolonged stable disease rather than tumour shrinkage [57]. The assessment of pharmacodynamic endpoints is another extremely important function for [^18^F]FLT. Scarpelli et al. [38, 39] demonstrated that the increase of [^18^F]FLT uptake is apparently caused by the washout period during treatment. This type of study, which evaluates the pharmacodynamic endpoints, is of great importance to develop better understanding of drug resistance mechanisms, as it helps clinicians to tailor more effective treatments and to minimise systematic toxicity caused by ineffective drugs [38]. Together, these preliminary findings suggest that [^18^F]FLT is a useful tool for evaluating response to targeted therapies with anticipated cytostatic effects, but further studies with different targeted agents, larger patient cohorts and longer follow ups are warranted to fully understand its potential in this role.

The studies mentioned above have multiple limitations. One limitation is that most studies are single-centre observational studies with small patient cohorts. This makes it difficult to know how readily the study findings would translate to other research centres or if similar results would be obtained using larger patient cohorts. While most studies used a relatively similar imaging protocol, there were no standard instructions for patient preparation. Some studies encouraged patients to drink water before imaging, which would help to reduce background radiation, whereas others did not. In addition, there were differences between studies in tracer dose administered, which is likely to affect the signal to background ratio. Most studies utilised static scans, with similar times between tracer injection and imaging. However, the use of different reconstruction techniques and metrics for assessing uptake (e.g. FLTmax vs FLTmean) would result in subtly different values being calculated from the same dataset. It is important that methods are optimised and standardised, so that the results obtained in one study can be directly compared with those from another, and so that multi-centre studies can generate equivalent data at all participating centres. This issue has been recognised within the PET community and has resulted in core labs being set up to ensure quality control for PET clinical trials [58]. The limited number of studies evaluating LC response to various types of treatment affects the conclusiveness of study findings. Moreover, size-based measurements were used as a standard reference in studies evaluating responses to targeted therapy although tumour size would not be affected with this type of therapy. OS was also used as the gold standard for assessing clinical outcome in studies evaluating responses to targeted therapy although it showed limitations compared with surrogate metrics such as PFS. Therefore, extra attention is also needed to ensure that the most suitable metrics of clinical outcome are used to study such therapies with [^18^F]FLT-PET.

## Conclusions

1. Overall, [^18^F]FLT seems to have better specificity in diagnosis and staging but lower sensitivity with significantly lower uptake than [^18^F]FDG. This significant difference was also observed after comparing uptake values of both tracers in the main histotypes of lung cancer that showed consistence with established histological patterns of cell proliferation in both tracers. Therefore, [^18^F]FLT cannot be considered superior to [^18^F]FDG for diagnosis and staging.
2. [^18^F]FLT uptake values of SCLC is considerably lower than SqCC. This indicates that SCLC may not be suitable for [^18^F]FLT imaging studies, due to limited radiotracer uptake.
3. [^18^F]FLT can provide good predictive values in patients undergoing radiotherapy or chemoradiotherapy and looks especially useful for assessing early response to targeted therapies.
4. [^18^F]FLT uptake values correlate well with TTP and PFS, but less well with size measurements based on computed tomography and OS.
5. [^18^F]FLT-PET tends to be promising in preliminary results of pharmacodynamic endpoints of targeted therapy predicting potential resistance which could permit better individualisation of treatment plans.

## Recommendations

1. More suitable reference standards such as histopathology for studies assessing response to targeted therapies are recommended for future studies.
2. TTP or PFS appear to be suitable clinical endpoints for [^18^F]FLT response assessment studies with targeted agents.
3. Studies should carefully consider which LC sub-type is recruited for imaging studies with [^18^F]FLT, as SCLC sub-type appears to have limited uptake.
4. More [^18^F]FLT studies are needed, to further evaluate the potential for this radiotracer to assess early response to therapy. This should include studies which evaluate different targeted therapies, studies which use standardised imaging protocols, multi-centre studies, and studies with larger patient cohorts and longer follow up times.

## Data Availability

The datasets used and/or analyses during the current study are available from the corresponding author upon reasonable request.

## Abbreviations

[^11^C]thymidine: ^11^C-labelled thymidine
[^18^F]FBAU: 1-(2-deoxy-2-[^18^F]fluoro-β-d-arabinofuranosyl)-5-bromouracil
[^18^F]FDG: [^18^F]fluorodeoxyglucose
[^18^F]FLT: 3'-deoxy-3'-[^18^F]fluorothymidine
[^18^F]FMAU: 2'-deoxy-2'-[^18^F]fluoro-5-methyl-1-β-d-arabinofuranosyluracil
ADC: Adenocarcinoma
EGFR-TKI: Epidermal growth factor receptor- tyrosine kinase inhibitor
LC: Lung cancer
LCC: Large cell lung carcinoma
MVD: Microvessel density
n: Number of patients
NSCLC: Non-small cell lung cancer
OS: Overall survival
PET: Positron emission tomography
PFS: Progression free survival
SCLC: Small cell lung cancer
SPF: S-phase fraction
SqCC: Squamous cell carcinoma
SUV: Standardised uptake value
SUVmax: Maximum standardised uptake value
SUVmean: Mean standardised uptake value
SUVpeak: Standardised uptake value peak
TK1: Thymidine kinase 1
TK2: Thymidine kinase 2
TNM: Tumour, nodal involvement and metastases
TTP: Time to progression
